# Inclusion strategies in multi-stakeholder dialogues: The case of a community-based participatory research on immunization in Nigeria

**DOI:** 10.1371/journal.pone.0264304

**Published:** 2022-03-22

**Authors:** Ngozi N. Akwataghibe, Elijah A. Ogunsola, Jacqueline E. W. Broerse, Adanna I. Agbo, Marjolein A. Dieleman

**Affiliations:** 1 Knowledge Unit Health, Royal Tropical Institute, Amsterdam, The Netherlands; 2 Faculty of Science, Athena Institute, Vrije Universiteit Amsterdam, Amsterdam, The Netherlands; 3 Ogun State Primary Health Care Development Board, Nigeria; 4 Amsterdam Public Health Research Institute, Amsterdam, The Netherlands; 5 Morgan State University, Baltimore, Maryland, United States of America; Universitat Luzern, SWITZERLAND

## Abstract

**Background:**

Community-Based Participatory Research (CBPR) has been used to address health disparities within several contexts by actively engaging communities. Though dialogues are recognized as a medium by which community members and other actors can make their voices heard through processes that support shared-decision making, power asymmetries often impede the achievement of this objective. Traditionally such relationship asymmetries exist between communities, health workers, and other professionals resulting in the exclusion of communities from decision making in participatory practices and dialogues. This study aimed to explore the experiences in the dialogues between different groups within communities, health workers and local government officials in a CBPR project on immunization in Nigeria. We adapted the framework by Elberse et al. (2011) to structure the possible exclusion mechanisms that could exist in dialogues between the three groups and we set up inclusion strategies to diminish the inequalities as much as possible.

**Methods and findings:**

This is an exploratory and descriptive case study, using qualitative methods. Data was collected through observation and semi-structured interviews (SSI) with dialogue participants. All 24 participants in the multi-stakeholder dialogues were interviewed. Inclusion strategies involved creating enabling circumstances; influencing behaviour; and influencing use of language. Verbal and circumstantial strategies were of limited value in reducing exclusion. Behavioural inclusion strategies created more awareness of the importance of inclusion; and enabled different community stakeholders to direct their influences towards achieving the collective goals of the collaboration. An important learning is that if evidence is used in the dialogues, even when exclusion of certain individuals occurs, the outcomes could still favour them. A key issue is the difference between participation and representation and the need for more efficient ways of carrying out such interactive processes to ensure that the participation of the vulnerable groups is not merely symbolic. The study makes a case for the use of ‘boundary spanners’ in this dynamic—these are ‘elite’ individuals (or community champions) who can be a voice for the minorities and who could have the opportunity to influence decision making.

**Conclusion:**

CBPR can enable local governments to develop effective partnerships with health workers and communities to achieve health-related goals even in the presence of asymmetries in relationships. Inclusion strategies in dialogues can improve participation and enable shared decision making, however exclusion of vulnerable groups may still occur. Intra-community dynamics and socio-cultural contexts can drive exclusion and less privileged community members require proper representation to enable their issues to be captured effectively.

## Introduction

Community-Based Participatory Research (CBPR) has been used to address health disparities within several contexts [1–4] by actively engaging communities [5]. There is evidence that CBPR has been effective across various cultural and disadvantaged settings through enhanced knowledge co-creation and exchange, and by reducing power imbalances [5, 6]. Wallerstein & Duran [5] found that CBPR created room for indigenous theories, culturally supported strategies and postcolonial knowledge. De las Neuces et al. [6] in their systematic review found that CBPR trials were highly effective in recruiting and retaining minority populations. A CBPR focused on maternal health services in post-Ebola Liberia improved community and health worker interaction and communication [7]. There have been several documented successes of the development and implementation of contextually appropriate health interventions at local level for communities via CBPR in other countries in Africa [8–10]. The three studies (two in Kenya and one in Zambia) involved participation of health workers and community members.

CBPR—frequently used synonymously with participatory action research (PAR) [11, 12]—emphasizes collective inquiry and research, based on experience and societal history [13] and broadly comprises a cyclical process of fact-finding, planning, action, reflection and re-planning [14]. A core principle is the development of meaningful partnerships with communities [5] and ensuring that marginalized populations have a genuine voice in the research [1]. CBPR emphasizes equitable partnerships and shared decision making between communities, professionals (experts) and researchers [10, 15]. These interactions often involve dialogues between these parties on topics of shared concern [16]. These dialogues are a two-way flow of information which involve mutual listening, sharing and questioning and enables communities’ views to be integrated as part of an iterative research process [17].

Despite the focus of CBPR on community participation and shared decision making, this is rarely realized in practice. This is because a power free space does not really exist in a social context. Though dialogues are recognized as a medium by which community members and other actors can make their voices heard through processes that support shared-decision making [18], power asymmetries often impede the achievement of this objective [19]. Cornwall and Jewkes [20] noted that the term ‘community’ encompasses a broad variety of actors who within their context are perceived differently and have unequal levels of power. Cranshaw et al. [21] stated that these unequal power relationships are amplified in CBPR involving health workers and communities because of socioeconomic and class differentials. In their study, which involved community dialogues in three contexts (Kenya, South Africa and Zambia), the authors observed that although the community custodians of social and religious customs and norms were not included in the dialogues due to fear of their presence stifling conversation, power differentials related to age, profession and gender still affected community participants [21]. Mercer-Mapstone et al. [19] also noted that addressing stakeholder participation from a top-down perspective was a driver of exclusion, particularly of minority groups.

Furthermore, several scholars have pointed at the difficulty of changing perceptions on knowledge as one of the main reasons for exclusion of less empowered community groups in CBPR. In addition to social status, asymmetry in relationships occur due to education and knowledge [3, 22, 23]. For instance, the (objective) knowledge of professionals is usually ranked higher by society than the more subjective (experiential) knowledge of communities [23, 24]. Elberse et al. [23] noted that this asymmetry is an obstacle to genuine partnerships between experts and end-users of health services (in our case, communities). They identified exclusion mechanisms which could hinder dialogues between experts, patients and their care givers and used inclusion strategies to encourage equal voice in decision making for all stakeholders [23]. The classic work by Arnstein [25] on citizen participation displayed the need for meaningful participation instead of ‘tokenism’. Mercer-Mapstone et al. [19] noted that authentic dialogues that result in a shifting of power dynamics in favour of those typically excluded from decision making processes requires actions of deliberate inclusion.

Many studies have acknowledged that the research and funding partners are usually at the fore of priority setting and decision making [3, 7, 26–29]. This exclusion of end-users of health services or communities from decision making in participatory practices and dialogues could be intentional or unintentional [23]. Elberse et al. [23] found that despite using inclusion strategies, there was still exclusion of caregivers and patients’ perspectives in the final outcome of dialogues held with medical professionals. They defined exclusion as “*the process whereby members of a certain stakeholder group or their perspectives are not taken up in the decision*-*making process*, *because of actions taken by members of other stakeholder groups or the process facilitator*.” [23; p227]. They described three categories of exclusion mechanisms during dialogues relating to the setting (circumstances), behaviour (what is done) and verbal communications [23]. The choices made relating to the period, place and length of time of a dialogue can result in exclusion of some participants [23, 30]. Similarly, differential treatment of participants in dialogues or formation of coalitions also result in exclusion of some. Furthermore, verbal communications that make it difficult for some participants to follow the conversations or which side-line the opinions of some are also important mechanisms of exclusion [23, 31]. A World Bank brief described socio-cultural values that hamper inclusion and facilitate exclusion within the African context and noted that identity was an important driver of social exclusion. Individuals and groups are excluded based on gender, caste, ethnicity, religion, age, occupational status, location, and disability status [32]. However, there is paucity of research relating to these exclusion mechanisms and the strategies used to ensure inclusion of vulnerable/disadvantaged groups in communities and their perspectives in decision making within stakeholder dialogues in CBPR within the African context.

The main objectives of this study are (1) to explore the experiences in the dialogues between different groups within communities, health workers and local government officials in a CBPR project on immunization in Nigeria in order to understand what exclusion mechanisms existed within the dialogues; (2) to investigate the extent to which the efforts to reduce exclusion of community women and men by the use of several inclusion strategies were effective, and (3) to explore how the strategies used influenced consensus building and decision making within the dialogues.

## Case description

In 2005, Nigeria adopted the Reach Every Ward (REW) strategic approach to improve routine vaccination coverage. According to the 2013 National Demographic Health Survey (NDHS), only 25% of children (ranging from 10% to 50% across geopolitical zones) aged 12 to 23 months completed a full course of prescribed routine immunization [33]. However, by 2015, Ogun state in South-West Nigeria had recorded consistent increase in routine immunization coverage across its twenty Local Government Areas (LGAs) with full coverage in twelve LGAs. Nevertheless, 16% of children (9,394) under one year were unimmunized across eight LGAs, with the highest burden in Remo-North LGA (37%).

A research consortium was established to develop and implement a CBPR project to address the problem of poor immunization utilization and coverage in parts of Ogun state. The study focused on Remo-North LGA. Two focal wards in the LGA were purposively selected based on immunization performance—assessed via the Health Management Information System (HMIS). In 2015, Ilara was the worst performing with immunization coverage of 26% and Ipara performed the best with a coverage of 75%. The wards also differed in other characteristics: Ilara is mainly a rural and remote farm settlement, located on the outskirts of Remo-North, while Ipara is semi-urban with a more organized structure and numbered streets. Each ward has one primary health care facility. In 2017, Ilara was less populated (6,512) than Ipara (9,100); and easier to navigate because the people live in clusters. The communal lifestyle of the people also makes for easy access to their king—the prime traditional ruler.

Situational analysis showed a history of collaboration between government, health workers and community members (especially indigenous groups) in both wards relating to health, education and other community development efforts. The Ward Development Committees (WDC) and the Social Mobilization Committees (SMC) are the official community social mobilization structures for immunization at ward and local government levels respectively. These structures are the official links to the REW strategy adapted by the state in 2006. The prime traditional rulers have considerable influence on these structures and on the collaborations. Traditional and political dynamics in the state reflect the patriarchal nature of the society with the community leaders (all or most of whom were male) and the male elders in the society having powerful roles in community decision making. For health interventions to be accepted and implemented, health workers and government have to first observe protocols of community entry through these traditional and community leaders. In the households, the men were also the primary decision makers on immunization issues, however, additional influences outside the nuclear family direct immunization utilization decisions. For instance, the grandmothers strongly influenced their sons; and the opinions and directions of the elders (grandfathers/fathers-in-law) were highly valued by the women.

The CPBR project took place between May 2016–April 2017. The project was implemented by a consortium comprised of the Ogun State Primary Health Care Development Board, the University of Ibadan, Nigeria and the Royal Tropical Institute (KIT), The Netherlands. The core research team was made up of three researchers consisting of a policy maker and two academics (one with quantitative and the other with qualitative background). They were coached on CBPR by two experienced qualitative researchers from KIT, The Netherlands. The researchers conducted the situational analysis in May 2016 and presented the results to the community members, health workers and the local government (LG) officials for validation. This evidence from the situational analysis informed the dialogues to develop joint actions. A pre-dialogue workshop was held with the three groups to discuss the inclusive aim of the dialogues.

### The dialogues

During the situational analysis, the vulnerable groups (such as migrants and young mothers) and key stakeholders relating to immunization in the communities were identified by the researchers. A guiding checklist based on the findings of the situational analysis was developed by the research team to ensure that relevant vulnerable and other groups within the context were all represented in the dialogues. The nomination checklist was presented to traditional rulers and community (including WDC) leaders and they nominated community members in their respective wards. LG management nominated frontline health workers and key LG stakeholders involved in immunization service delivery. The checklist used for the nomination of community members included: Christians, Muslims, at least one traditionalist, at least one foreign national from Cotonou (Benin republic), at least one person from the Igede (migrants), five young (under 40 years) and five older women per ward, five young and five older men per ward, and representatives of community social mobilization structures (ward development committees and social mobilization committees).

The nomination process aimed to ensure the inclusion of identified vulnerable groups and equipped participants within the community (in terms of links to and knowledge of immunization as well as representation of their various groups). We were aware that this process could lead to the inclusion of people who may be more reticent in expressing their opinions or who may not be as motivated as others, but we considered the involvement of all the relevant stakeholders critical since the principle of inclusion is key to participatory inquiry.

Single stakeholder dialogues took place first—Community members per ward, health workers in their respective wards and Remo-North LG officials. For the community members in each ward, the women held their dialogues separately from the men; and each group then nominated representatives for the dialogues between community men and women. The participants in the dialogues had been nominated by community leaders (for community members) and the LG authorities (health workers and LG officials) and accepted the nominations voluntarily. Community members who considered that they would not have time for the dialogues could reject the nomination. Research assistants helped in the facilitation of the dialogues—mainly as observers and note-takers (for the action plans in the community dialogues).

**Fig 1:**
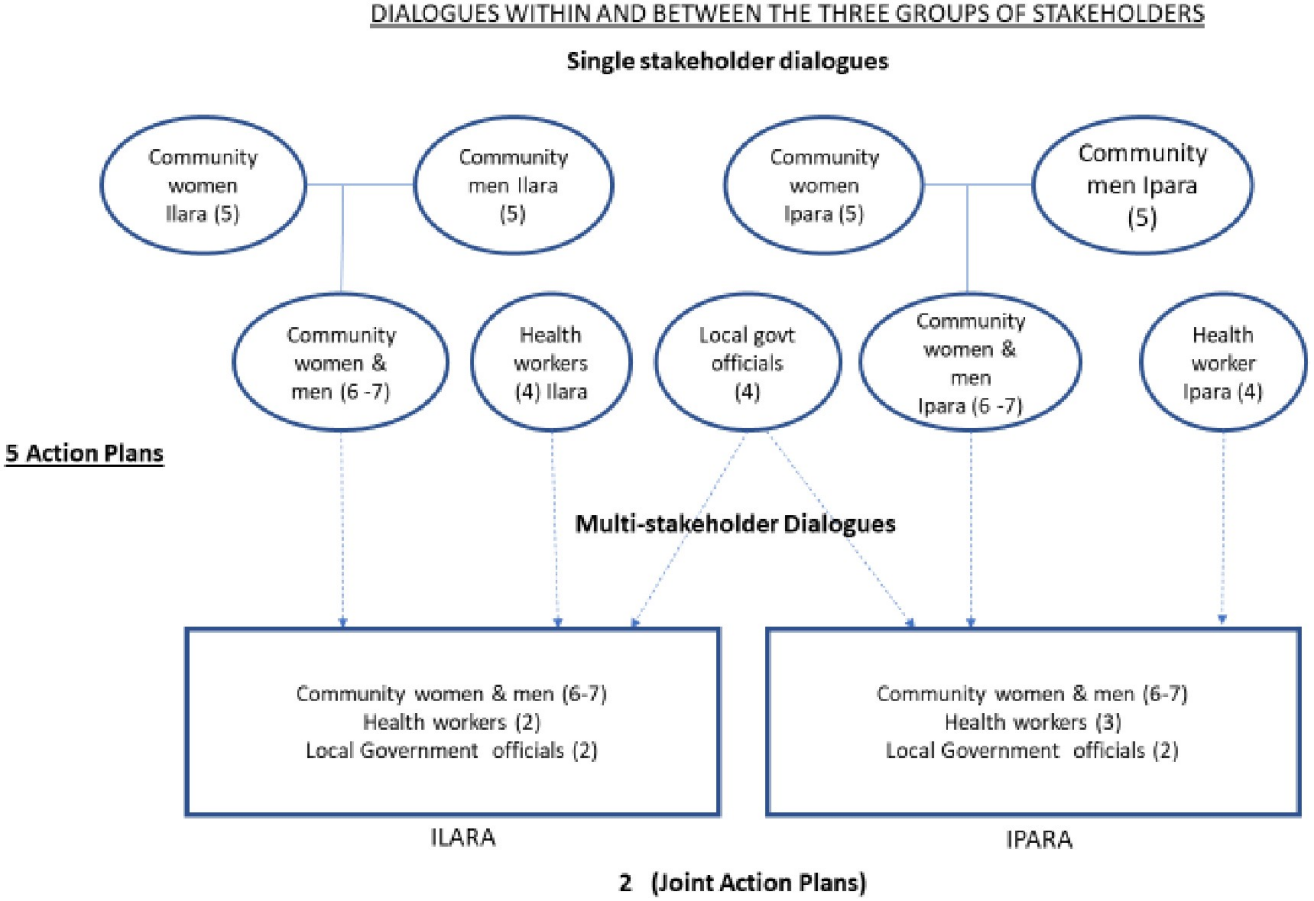
Single and multi-stakeholder dialogues.

For the multi-stakeholder dialogues a structured process was followed. After the discussions on actions by each stakeholder group, nominations for the multi-stakeholder dialogues were made by consensus of members of the individual groups. The research team tried to manage the asymmetry in the relationship between the three groups of stakeholders and encouraged that six/seven community members per ward were nominated; two health workers in Ilara and three in Ipara were nominated; and LG nominees were only two per ward. The LG officials had more authority and influence than the rest of the groups and it was uncommon that they would sit together with community members to discuss issues. The health workers—though also in a position of influence due to their official mandate of health and immunization service delivery—were more familiar with the community members. To lessen feelings of intimidation and add some leverage to the community members during issues that required voting to arrive at a decision in the multi-stakeholder dialogues, the number of community members was higher than those of professionals (6–7 versus 4–5, respectively). In each ward, the actions and plans formulated per group were compared and discussed within the joint dialogue groups to develop Joint Action Plans (JAPs) for change. After the multi-stakeholder dialogues, the participants who now referred to themselves as the Joint Action Committees (JAC) and implemented the JAPs in both wards over an eight-month period. Fig 1 illustrates the single and group dialogues. A summary of the results of the single group dialogues is in S1 File. This article focuses on the multi-stakeholder dialogues.

Considering the asymmetry in social status, education, and knowledge between the three groups, we tried to identify possible ways that community women and men could be excluded from influencing decision making. We used Elberse et al.’s [23] framework to structure the possible exclusion mechanisms. We intentionally set up some inclusion strategies to diminish the inequalities as much as possible. We present these strategies in Table 1 below. The inclusion mechanisms were also informed by Elberse et al. [23].

**Table 1:** Possible exclusion mechanisms and the inclusion strategies used in the dialogues of the CBPR project.

Category	Possible exclusion mechanisms	Applied inclusion strategies
**Circumstances**	Uncomfortable location for patients, uncomfortable setting	Neutral venue accessible to all parties, away from the influence of traditional leaders and elders, where community members would feel free to express their opinions (i.c. a town hall, sitting arrangements, no function tags)
	Health workers and LG officials outnumber community members	More community members than health workers and LG officials were nominated aimed at achieving power in the numbers. It was expected that outnumbering government /health workers would contribute to reducing the asymmetrical power relations because the community members will feel more supported by each other to speak up during the dialogue
	Unfamiliar with working methods	Clear explanation and instruction were given to participants on what to expect and the aims of the CBPR, as well as the need for everyone to listen and feel listened to
	Choice of focus and scope	The dialogues were designed with a focus on capturing the separate perspectives of the community men and women, health workers and local governments followed by a general focus on the whole group and a shared action and implementation plan
**Behaviour**	No opportunities for community members to speak	The dialogues provided equal opportunities to speak. Facilitators were instructed to stimulate input of community members, especially women
	Health workers and LG officials stress their ‘elite’ positions and intimidate community members	Pre-dialogue workshop was held with the three groups to discuss the inclusive aim of the dialogues and action process and the need for community ownership in order to develop and implement sustainable local solutions; the power asymmetry between the three groups and the need to ensure that community members had equal opportunities to speak and felt listened to. Stakeholders were encouraged to develop a memorandum of understanding (MoU), which would be adhered to by all. Stakeholders were encouraged to select community members as chairs of the multi-stakeholder dialogues.
	Socio-cultural norms and gender relations result in the women not speaking	Single stakeholder dialogues with young and older women to capture views before dialogues with community men to develop the community action plans. Facilitators instructed to stimulate women to speak.
**Verbal**	Use of jargon	Health workers and LG officials were requested to use plain language.
	Ridiculing the opinions of community members; Side-lining community members’ issues as not relevant, not feasible, etc	Pre-dialogue presentations emphasised the importance of the community members’ perspectives to all three groups of stakeholders. Research assistants were instructed to guard the input of the community members and ensure that they were captured accurately in the community and joint action plans. Stakeholders encouraged to develop MoU.
	The use of English language in multi-stakeholder dialogues may result in community members not speaking	Dialogues involving the community members were all held in the local Yoruba language

## Methods

This exploratory and descriptive case study, using qualitative methods, addressed the question of how the use of inclusion strategies influenced consensus building and decision making in the multi-stakeholder dialogues. More specifically, (1) How inclusive were the multi-stakeholder dialogues? (2) How was consensus built—were the perspectives of all the stakeholders included or did exclusion take place—if so, how and for whom? (3) How did different people use their influence to change directions and affect decisions in the dialogues? And (4) Did the inclusion strategies result in the needs of vulnerable groups being taken into account in the decision making?

### Data collection

Data was collected through observation and semi-structured interviews (SSIs) with dialogue participants. During the dialogues, observational checklists were used to capture information on who started a new topic, dominated the discussions, proposed solutions, made decisions, disagreed with decisions, etc. With the SSIs, we captured information on the perceptions of the dialogue participants regarding the interactions and negotiations that took place; the basis on which decisions were taken; and perceptions of the community members of their inclusion in the dialogues and decision making. The topic guides for the SSI are in S2 File. All participants in the multi-stakeholder dialogues were asked to be interviewed by the research assistants. Interviews and observations were carried out by an experienced qualitative researcher and eight trained qualitative research assistants.

All 24 participants in the multi-stakeholder dialogues (12 in each ward) were interviewed. 16 out of the 24 JAC members interviewed were female. Tables 2 and 3 present tables of the JAC members in Ilara and Ipara respectively, detailing their gender, occupations, and JAC functions. The groups in the wards selected a chairman (a community member) and a secretary (health worker) chosen for literary and technical skills. They also nominated women leaders responsible for leading the mobilization of young women (caregivers). In Ipara, more of the older community women were chosen to dialogue with the men. Both wards aimed at ensuring that the more vocal women in the groups represented them—usually women that had leadership roles in the community and were used to such interactions with men, such as the Traditional Birth Attendant (TBA) in Ilara. However, some young women were chosen in Ilara, since immunization was considered more relevant for this group. The multi-stakeholder dialogues were facilitated by the JAC chairmen and proceedings were recorded in writing by the JAC secretaries in both wards.

**Table 2:** Ilara JAC members.

Sn	Ilara JAC members	Age (Years)	Sex	Occupation
1	Community Member (WDC/JAC Chairman)	45	M	Clergyman / Farming
2	Community Member	27	F	Hairdressing
3	Community Member	NA	M	Native Doctor
4	Community Member	32	F	Trading
5	Community Member	47	M	Security Man / Farming
6	Community Member	NA	M	Farming
7	Health Worker (JAC Secretary)	38	F	Health Worker
8	Facility Health Worker	57	F	Health Worker
9	Traditional Birth Attendant (JAC Women Leader)	69	F	Health Worker
10	Principal Medical Officer of Health	44	F	Local Government Official
11	Health Worker in-charge / ward focal person on immunization	45	F	Health Worker
12	Disease Surveillance Officer	38	F	Local Government Official

**Table 3:** Ipara JAC members.

Sn	Ipara JAC members	Age (Years)	Sex	Occupation
1	Community Member (WDC Chairman)	57	M	Retired Teacher
2	Community Member	42	M	Clergy / Farming
3	Community Member	69	M	Clergy / Farming
4	Community member (Alhaja / JAC Women Leader)	NA	F	Trading
5	Health Worker (matron-in-charge) (JAC Secretary)	53	F	Health Worker
6	Health Worker	NA	F	Health Worker
7	Health Worker	42	F	Health Worker
8	Community Member	43	M	Trader / Farmer
9	Local Government Immunization Officer (LIO)	53	F	Local Government Official
10	Community Member	42	F	Trading
11	Community Member	48	F	Trading
12	Health Educator	55	M	Local Government Official

### Data processing and analysis

The observation checklists were analysed to understand how the groups worked together; and relational dynamics between the three groups. At least two research assistants observed each dialogue session. The observation checklists were compared, and areas of disagreement discussed for clarity. The interviews were audio-taped and transcribed verbatim by research assistants. Interviews conducted in Yoruba language were transcribed and translated to English.

The qualitative data analysis was carried out using NVivo 11. A thematic approach (identifying emerging themes through coding and labelling qualitative data) was followed. Transcripts were read by two researchers, coded and common themes and sub-themes identified according to the research questions, the adapted framework, topic guides and issues emerging from the transcripts. A codebook was developed with an outline of the themes, sub-themes, codes and code descriptions. A third researcher ratified these by using the codebook to code a few transcripts. All three researchers had earlier conducted an initial systematic and independent reading of the transcripts. Observational notes were analysed by one researcher and discussed with a second researcher. The SSIs were analysed using a three-pronged approach: “noticing, collecting, and thinking” [34]. We analysed how consensus was built and how this had been influenced by the inclusive strategies set up in the project. We also analysed how stakeholders used their positioning, social status and authority to influence decision making and the direction of the dialogues; and whether there was exclusion of any stakeholders. We triangulated with data from the SSIs to see if our observations matched with the perceptions of the stakeholders.

### Ethical considerations

Ethical approval was obtained from the University of Ibadan, University College Hospital Ethics Board (UI/UCH Ethics Committee assigned number UI/EC/15/0447) and the World Health Organization Ethics Review Board. Verbal and/or written informed consent was obtained from all the study participants—depending on the literacy level and choice of the participant. Privacy and confidentiality was assured for all respondents. We delinked personal identifiers from data and we used appropriate venues for the interviews. Additionally, permission for the study was granted at three levels—the state Primary Health Care Development Board (SPHCDB)/Ministry Of Health, the Remo North LGA and from the Community through established Community Development Associations.

## Results

We explore how inclusive the multi-stakeholder dialogues were in relation to circumstances, behaviour and verbal exclusion mechanisms using the adapted framework of Elberse et al. (2011). Where issues overlap, they are reported mainly in one section. Many of the issues relate similarly to both wards but where there are differences, these are highlighted. Prior to the dialogues, the process was explained to the three groups by the researchers and discussions were held about the process for consensus building. The strategies to address circumstances and verbal mechanisms resulted in some facilitation of inclusion but only marginally. The main issues were related to the behavioural component.

### Circumstances

Several strategies were put in place to encourage inclusion of community members in decision making. These included making sure the community members outnumbered the professionals; clear explanations on the participatory approach; and setting up single-group dialogues to ensure that the perspectives of the stakeholders were captured before the multi-stakeholder dialogues (see Table 1).

The initial plan had been for the dialogues to be held in venues within the respective wards. However, to encourage more openness by the community members in voicing their views, a neutral venue in a neighbouring town—Ode—was later chosen by the research team for the dialogues. This was considered an accessible location by participants in both wards and transportation costs were reimbursed. Nonetheless, a few Ipara community participants complained about the distance and inconvenience. The Ode Town Hall was used and the table settings were kept neutral—there were no differences observed between where the health workers, LG officials and community members sat. Name and function tags were not used as most people in each ward were familiar with each other. Overall, the use of a neutral venue did not prevent the feelings of exclusion some community members could have felt—some were still reticent and spoke only when they were assured that issues were not controversial.

#### Set up of stakeholders and consensus building

JAC members from the three groups described a democratic process of consensus building and decision making in the multi-stakeholder dialogues. Prior to voting, discussions were held to try and reach consensus. Voting was conducted on different issues and majority votes on decisions were carried forward as group decisions. The increase in the number of the community members may have provided an advantage and enabled communities’ perspectives to be captured, but this was not explicitly reported by the respondents. However, the voting process was described by many participants in Ipara:

“*It is important that we agree because the majority carries the vote*. *Whatever we have collectively agreed on*, *nobody should be left behind*. *This is in our laws (MoU) which we formulated ourselves*.”

– *Community Member (Clergy*/*Farmer Ipara)*

Before final decisions were made through voting, participants discussed and negotiated on issues. All three groups of stakeholders reported that consensus was built after deliberations and elaborate discussions. Arriving at decisions were typically described as not taking too long (time given was from 1–3 hours). Increasing the numbers of the community members in the dialogues may have boosted their influence when issues were tabled for individual voting, but this advantage appeared to be limited. Nevertheless, relative ease was described in reaching most decisions, including prioritizing items on the JAPs. It may have helped that the topic was not such a controversial one—the importance of immunizing young children was regularly mentioned by all the parties.

Though the dialogues were reported as free from conflicts and issues were generally resolved easily, some topics resulted in more arguments than others. Participants in Ipara especially but also in Ilara, described situations where they ‘agreed to disagree’. One issue that seemed to cause a lot of arguments was money. Participants in Ipara described this more frequently than their Ilara counterparts:

“*When someone says his idea and the group knows that it might involve money*; *issues like this might cause shouting at first*. *We will argue it to the left and right*. *Then we settle after we calmly talk about the importance of money*.”

– *Community member (Trader*, *Ipara)*

Indeed, JAC community members in Ilara did not mention arguments regarding money—yet many had contributed financially towards the JAPs. It is possible that to ease consensus building on issues, participants had deliberately avoided friction relating to discussions on money.

#### Explanations on the participatory approach

Presentations and discussions had been carried out with the three groups of stakeholders before the dialogues. Explanations and instructions had been given to participants on what to expect and the aims of the CBPR, as well as the need for everyone to listen and feel listened to.

Community members, health workers and LG officials in both wards all mentioned that they were able to contribute to the dialogues—that they listened to others and felt listened to. However, despite the explanations given at the start of the CBPR, some respondents had the opinion that a few other participants had not fully understood the participatory process at the start of the dialogues though more clarity had occurred as the discussions had progressed. This did not appear to influence the dialogues. Many respondents perceived that the comfort levels in expressing their views and willingness to speak had noticeably increased during the course of the multi-stakeholder dialogues. This was reported more for the Ilara than the Ipara participants.

#### Choice of focus and scope

The dialogues were designed with a focus on capturing the separate perspectives of the community men and women, health workers and local governments followed by a general focus on the whole group and a shared action and implementation plan.

Comparing the identified problems by the different groups of stakeholders in Ilara with the final negotiated JAP showed that all the priority problems identified by the local government officials and almost all identified by the health workers were captured in the JAP. While most of the issues identified in the community action plans were prioritized in the JAP, some were left out. This may have been because they were not considered feasible during the multi-stakeholder dialogues. The identified issues left out of the JAP were: non-availability of a pharmacy at the health center; poor accommodation for health workers and poor road network to Ilara. In contrast, In Ipara, almost all the problems identified by the community members were prioritized in the negotiated JAP and most but not all the identified issues by the local government officials and health workers were captured.

#### Influence of traditional structures

Overall, cultural, social, political, and economic powers of the traditional rulers exerted strong influence over decision making, even though the traditional rulers were not directly involved in the dialogues and the neutral venue away from the wards had been selected to enable all community members participate freely in decision making without interference from local political and traditional leaders. However, some of the health workers and local government officials in Ilara believed decisions made during the dialogues would only hold if the Kabiyesi (the foremost traditional ruler of the ward) did not disagree with them. The Kabiyesi had strong authoritative powers and respondents emphasized his support as being critical.

“*The decisions that we cannot make immediately is left for the king*; *we need the approval of the king in whatever we do*. *He is not directly involved but it is his own community*. *Whatever we discuss here*, *some of his people are there so he hears about it and if we are taking a decision that is not right for his community*, *he may say* “*No*”. *So there is nothing we can do*, *because he owns his community so he can take decisions*”.

– *Health worker 2 (Ilara)*

Participants gave some examples of barriers to implementation of the decisions made in the dialogues (such as advocacy for funding from philanthropists within the wards) because of delays due to traditional protocols:

“*Even though you know a philanthropist on your own*, *you have to go through the king*. *It*’*s their tradition*. *And I told you before that their culture*, *their beliefs and values*, *standards must be respected*… *However*, *going through the king*; *it is a big problem*. *You know*, *sometimes the king is not around*…”

– *LGA Official 1 (Ilara)*

It was not obvious from the interviews how the dynamics between the community members who had access to the kings (such as the JAC chairmen, WDC members, some older men and women) and those who did not have access played out. From the interviews, it was clear that the deliberations in the dialogues, especially in Ilara, were discussed further with the traditional ruler by this privileged group. This suggests that the views of the community members that would be prioritized in those discussions may depend on the interests of these individuals.

### Verbal inclusion

The strategies used to encourage behavioural inclusion also relate to verbal inclusion especially the pre-dialogue discussions. In addition, health workers and LG officials were encouraged to avoid the use of jargon and to communicate in the local language during the dialogues (see Table 1).

#### Avoiding use of jargon and sidelining of community participants

None of the participants reported that their input during the dialogues were ridiculed. Many of the participants referred to the MoU; the ground rules had been set by the participants before the dialogues started, regarding procedures and how they would respect each other’s rights to talk and contribute. This appeared to have been taken seriously by all the participants. Nonetheless, the issues regarding verbal exclusion appeared to be more covert, and may have resulted in the side-lining of some community members’ views as not feasible as described in the previous section.

#### Language

The official working language for the LG officials and health workers is English but the local language was used during the dialogues and action. The use of the Yoruba language was perceived to have helped to put many community members at ease and encourage their willingness to talk during the dialogues. This was perceived by respondents from all the groups as having enabled meaningful participation of the community stakeholders by easing verbal expression of opinions. This also applied to the vulnerable (non-indigenous) groups, many of whom had been born there or had lived in the communities for a long time. Those that were more fluent in the Yoruba language had been chosen for the dialogues and action.

“*We conduct our meetings in Yoruba so for everybody*, *it is convenient*. *Even the non*-*indigenous hear Yoruba because some of them are born here*. *Some have spent more than 30 years*. *They may not be able to speak fluently but they understand and when they speak*, *we pick their ideas*.”

– *JAC chairman (Ipara)*

### Behavioural inclusion

Several strategies were applied to encourage behavioural inclusion (see Table 1). These included: working methods that provided equal opportunities to speak and stimulating input of community members especially women and pre-dialogue explanations of the inclusion aim of the dialogues. Additionally, single stakeholder dialogues (not reported in this article) had also been held prior the multi-stakeholder dialogues to ensure that all the perspectives of the three groups of stakeholders were captured.

#### Equal opportunities to provide input

Though the developed MoU and the facilitators encouraged all the community members especially women to speak during the dialogues, gender and age-related issues sometimes led to exclusion of some individuals. According to some respondents, during the dialogues, the Ilara young women especially were reticent, probably due to the social norms of keeping quiet before their elders and men. A female LGA stakeholder described having to intervene to ensure that the young women could speak during the multi-stakeholder dialogues:

“*We were having a meeting and I just observed the younger women were not talking*, *I had to tell them*, “*you should talk*”; *but you know in our tradition women don*’*t just talk anyhow*. *It is only those of us that are elites that can talk anyhow*. *I asked the men*, “*Men in the house*, *are you telling these women not to talk*?” *The said* “*no*, *they can contribute*” *and immediately the young women started contributing*.”

– *LGA Official (DSNO Ilara)*

The initial reticence was expressed frequently as being noticeably less during the second round of dialogues. This was in line with the observations. Age also appeared to be a factor here; in Ilara a few young women had been chosen to represent the community groups in the multi-stakeholder dialogues, and they described age-related social norms:

“*I think it is because they do consider that they are elderly persons and that their ideas should be considered before the younger ones ideas will be looked into*.”

– *Hairdresser (Ilara—27 years)*

“*Whenever we all sit for a meeting*, *if someone speaks up*, *another person would say their opinion and then the elderly ones among us would get the best of decision out*.”

– *Community member (Trader—32 years)*

The community female group in Ipara had chosen only older women to represent them in the multi-stakeholder dialogues and did not report this barrier. Older women in the communities appeared to be more influential—for instance, the TBA in Ilara and the Alhaja in Ipara both had access to their kings.

Although some women’s views were given weight during decision-making, because of their knowledge or position, overall men seemed to have more influence on decision making. The JAC chairmen in both wards were acknowledged by other stakeholders, including the LGA officials, as highly influential in decision making. The JAC chairmen also reported using their authority to provide direction to the dialogue proceedings and control who was allowed to talk (usually described within the context of ensuring order to the proceedings).

“*Even if some people try taking it personally*, *we will tell such a person to be calm*, *because we made it clear that it is only those who the chairman gives chance to talk*, *that will talk*. *When an issue is raised*, *we don*’*t allow anybody except the person that raised the issue to talk and explain*, *before we give room to someone else*. *And if we see it*’*s an issue that can cause chaos*, *we will suspend it and talk about something else*.”

– *Chairman JAC (Ilara)*

“*They have no choice because I direct the meeting they have to respect my opinion and I respect theirs too*. *Respect begets respect*.”

– *Chairman JAC (Ipara)*

It seemed a natural turn of events for men to chair the dialogues within the patriarchal contexts and for the government officials and the health workers to leverage on the respect given to men in order to achieve set objectives. This was not seen as out of place by many participants as shown in the conversation below:

*I*: “*Are there people present in the group who are much more influential than others*.”

*R*: “*Yes*, *there are*. *There is no group where you would not have capable people*, *it is not as though they are commandeering in their approach but people do listen to them whenever they talk*.”

*I*: “*Who and who are those people*.”

*R*: “*Men*.”

– *Igede representative (Ipara)*

#### Pre-dialogue explanations of the inclusive aim of the dialogues

The pre-dialogue workshop held with the three groups highlighted the asymmetrical relationships between the groups and the need for inclusion of all parties in the dialogues and action process. The need for equal participation of community members in decision making and community ownership in order to enable the development of sustainable local solutions was emphasized by the research team. The three groups of stakeholders developed the ground rules and an MoU to enable contributions from all the parties involved. Additionally, they selected community members as chairs of the multi-stakeholder dialogues to empower the community stakeholders in view of the social /professional status of the health workers and LG officials.

We examined how all these had worked to enable behavioural inclusion and noticed three main types of influences, which controlled the directions and decisions in the dialogues: influences due to (1) experience and knowledge; (2) hierarchy and status in society; and (3) cultural norms and values.

#### Influence due to experience and knowledge

Analysis of the observation checklists showed that contributions to discussions were partly determined by people’s experiential and technical knowledge—for instance, the health workers and LG officials had more knowledge of the health and immunization system; the Igede representatives contributed more to issues relating to their ethnic group; the JAC chairman in Ipara contributed a lot on the immunization work done by the WDC. The older women also highlighted views based on their experiences as community links to immunization services. It appeared that the views of the community members were considered useful by the LG officials and health workers, but the weight or value placed on these views may not have been so much as exemplified by the quote below:

*I*: “*Do certain individuals have more influence over the agenda at group meetings than others*?”

*R*: “*Hmmm … yes*. *Like in our group*, *there is one man*. *As young as he is*, *he gives you good ideas and I can see he is influential in the community*. *So when you give suggestions*, *he tells you that cannot work*. *And you cannot just talk like that if you are not someone that is notable in the community*.”

*I*: “*So he has more influence …*”

*R*: “*Not that much … but he knows virtually everything about the community…*”

– *LG Official (IIara)*

Usually, the local government officials and health workers were regarded as the knowledgeable ones and many community members expected that they should be more influential in decision making.

*I*: “*Sir*, *within these groups we have mentioned*, *who are those who make decisions*?”

*R*: “*It is often left to someone who has the most understanding*. *Such people know the right things to be done at the right time*, *and who will know how to tell the others*. *The health sister*’*s ideas are important because we know she is one of the enlightened ones*.”

– *Community member (Native (traditional) doctor*, *Ilara)*

#### Influence due to hierarchy and status in society

Several people were recognized by participants as playing leadership roles during the dialogues—and taking charge of the discussions. These included the chairmen of the JAC in both wards, health workers—specifically the ones who were the ward focal persons on immunization, the TBA in Ilara and the local government Principal Medical Officer of Health (PMOH). Many community members (especially in Ilara) considered that some people by virtue of their functions and hierarchy were more informed than others and therefore in a better position to make decisions.

The choice of community members as JAC chairmen was an inclusion strategy. However, it was clear that they were chosen by the participants because of their status in the society. Both chairmen were frequently described as influential by the three groups of stakeholders. Indeed, it was evident that some community members were regarded as ‘elites’ even by the LG officials and this gave more weight to their opinions and actions.

*R*: “*There are some members of the community that will cheer you up*. *So of course they influence you and you respect them*.”

*I*: “*Why do you think this is easier for those people*?”

*R*: “*Because of their class*.”

– *LG (DSNO*, *Ilara)*

While it was apparent that the JAC chairmen played important roles in the direction of the dialogues, this did not necessarily improve inclusion of other less influential community members. Nevertheless, the LGA officials appeared to leverage on the influential positions of these key community stakeholders:

*I*: “*Do certain individuals have more influence over decision making process than others*?”

*R*: “*The Chairman has more influence*, *he can persuade them over an issue to do it*.”

*I*: “*And what do you think about this Ma*?”

*R*: “*To achieve our goals*, *I think it*’*s good—not that he is imposing on them—but he encourages everyone so that they can achieve the set plans*.”

– *LGA Official (PMOH*, *Ilara)*

LGA officials seemed to use their hierarchical position within the health system to provide some direction and leadership to the dialogues, but they were intentional about ensuring the involvement of the community members in the discussions and decision making. Their descriptions frequently displayed their views about ‘allowing’ the community members to contribute.

“*I understand the community a lot*. *You don*’*t give them suggestions*, *you let them bring out suggestions and you help them reshape it*. *It is not that I don*’*t bring out suggestions but we allow them make more contributions than we do*.”

– *LGA (DSNO*, *Ilara)*

#### Influence due to cultural norms and values

Participants described the diversity of those who had been included in the dialogues—different community groups were represented including traditional rulers, religious leaders, and different tribal groups. The LG officials appeared to have leveraged on this diversity intentionally to enable ease of the proceedings.

“*What we did was is if we had any challenge with any group we brought them into the committee (JAC)*, *because once they are in it will be difficult for them to work against the interest of the committee*.”

– *LG Official 3 (Ipara)*

In both wards community members with religious, social, cultural, and (sometimes) economic influences appeared to feel empowered in the negotiations by virtue of their standing in society. The tones in which they described their participations in the negotiations often differed from those of other community members who were of the view that certain individuals within the groups were better informed and therefore better placed to lead and direct the negotiations. In Ipara community members with this social status included the JAC chairman, the Christian Association of Nigeria (CAN) representative and the women leader (Alhaja). In Ilara, the JAC chairman and the TBA (women leader) were the most influential. Community members who held influential religious and social positions within the society claimed their authority to take decisions because of these positions. The quote below is an example:

“*I know they carefully listen because nobody will say* “*Shut up*, *you speak nonsense*” *when they know the people I*’*m representing*. *They calm down to hear what I have to say and deliberate on it*.”

– *CAN representative (Ipara)*

The perception of some people in the group being more influential in decision making were not viewed as out of place. Many of the community respondents considered this a way of life.

“*That*’*s how God designed things*, *someone must be the leader*.”

– *Alhaja (women leader*, *Ipara)*

Some of the community members, with less standing and resources, felt indeed less influential:

“*You know*, *when you say something and they didn*’*t make use of it*, *you will know*. *When I say something*, *they will be looking at me as if they are not interested and when I finish talking*, *they will cut in and say* ’*are you through with your talking*?”

– *Community member (Hairdresser*, *Ilara)*

Indeed, views expressed by some gave the impression that they considered themselves a bit removed from the proceedings even while they claimed active participation.

“*They try as much as possible to listen and adjust to the complaints of the society*. *On my own end*, *I participate*. *They might disagree with each other but they are always ready to listen*.*—Community member*”

– *Security Man* / *Farmer (Ilara)*

In essence, though a democratic process of consensus building and of decision making had been followed, local government, health workers and some community members in the dialogues used their different influences to control decisions and the directions of the dialogues and actions. Many of the JAC community members had social, religious or political influences, which were leveraged on for issues on community mobilization and awareness creation on immunization; health workers and LG officials had official authority, knowledge on immunization and control over information; LG officials also had policy influence. It was obvious in the observations that all these were used in concert to influence the development (and execution) of the JAPs.

### Influence of exclusion on outcomes (the JAPs)

The question was then: How did all the issues relating to circumstantial and behavioural exclusion influence the results of the dialogues—especially since certain perspectives may not have been voiced by some vulnerable groups? To gain some insight, we compared the problems identified during the single stakeholder dialogues and the final JAPs developed after negotiations. We found that interestingly, key issues relating to the vulnerable groups had been captured and prioritized in the JAPs. It appeared that using evidence from the situational analysis in the discussions had enabled the stakeholders to keep those issues in view. Though certain vulnerable groups seemed to be out of the loop in decision making, their interests appeared to have been represented by stronger stakeholders especially the LGA officials. For instance, the LGA officials ensured that the actions related to creating more awareness of the usefulness of immunization in the communities also specifically targeted the migrants. During implementation, the committees ensured that the migrants within the JAC led the discussions with their communities.

It was also noticeable that more prioritized actions were reflected in the Ipara JAP for the prioritized problems of the community members than was seen in the Ilara JAP. The priorities not chosen for Ilara during the multi-stakeholder dialogues (some of which were also highlighted in the LGA plan) were considered not feasible due to lack of resources. It is possible, that for Ipara, the representation of community members—many of whom were more educated and had strong alliances with powerful groups (WDC, CAN etc)—enabled the identification of more feasible priorities and stronger community negotiations.

## Discussion

This article offers insights into if and how inclusion strategies worked in community dialogues within the framework of participatory research. It also highlights exclusion mechanisms that can emerge when communities, local governments, health workers (facilitated by researchers) are engaged in dialogue meetings. It contributes to the CBPR literature by showing how local governments can develop effective partnerships with health workers and communities to achieve health-related goals amid asymmetries in relationships. We used several inclusion strategies (creating enabling circumstances, influencing behaviour and influencing use of language) to address relationship differentials. However, it is obvious that a power-free space within the societal context is not possible—exclusion still occurred within the socio-cultural environment and was related mainly to behaviour.

### Positive effects of inclusion strategies—i.e. what worked well

In addition to underscoring the role of inclusion strategies in CBPR interactive processes, this study showed the importance of being aware of (and transparent about) asymmetry in relationships. In our CBPR, this enabled LG officials and health workers to be more intentional about sharing decision-making; and community members to be more intentional about taking responsibility for action and displaying action-oriented behaviour. Channelling stakeholder influences towards producing positive outcomes proved useful. For instance, the role of the foremost traditional rulers was recognized and taken into consideration by the LG officials. It is expected within the context to exercise respect for the traditional protocols to ensure uptake and acceptability of interventions by the communities. However, it is pertinent to highlight that this may concentrate decision making in the hands of a single individual and stifles participation.

The dialogues provided a conducive space for people in the three groups (who would not normally have the opportunity) to talk to each other about important health systems issues. This enabled the establishment of new interactions between the stakeholders and initiated trust-building. This presents an opportunity for developing better ways of working together. The deliberate use of the local Yoruba language in the dialogues by the LG officials and the health workers contributed to reductions in the relationship asymmetries. Wallerstein and Duran [5] indicated that it is a sign of greater partnership if people can communicate in their own language. They described this as a type of cultural humility exercised by researchers (and in our case, health workers and LG officials). Cultural humility is described by Tervalon & Murray-Garcia [35] as a commitment to address power asymmetries by maintaining respectful and vibrant partnerships with communities. Nevertheless, verbal strategies and those related to circumstance were not sufficient to counter the power imbalances in the behavioural domain.

Behavioural inclusion strategies enabled different community stakeholders to direct their influences towards achieving the collective goals of the collaboration. The dialogue participants were more aware of the importance of inclusion and that was a step towards being inclusive. The pre-dialogue workshop was shown to be an effective strategy in this arena—especially enabling LG and influential community stakeholders to keep the inclusion aim in view throughout the dialogues. Female gender influences in decision making were shaped by official authority (LGA female officials) and local political / traditional structures and age (older community women leaders). Though not a deliberate strategy in the study, the influences of these women were leveraged to address community level issues.

### Exclusion observed and its effect on the dialogues

Elberse et al. found that exclusion of care users’ perspectives still occurred in their dialogues with experts despite implemented inclusion strategies [23]. Similarly, in our study, there was exclusion of some community members from joint decision making. Gender and age norms appeared to tilt more decision-making influences towards the men and older persons respectively. There was evidence of exclusion of the young women (young mothers) for whom the issues were most relevant, raising questions about how much of their perspectives were reflected in the JAPs. In the interviews, a few young women referred to the rest of the group (especially older ones and those with greater social status) as “they”—thereby painting a picture of distance between them and the rest of the group. The presence of the LGA female officials, who had double roles (gender/authority), ensured that the relevant female perspectives were captured in the JAPs. Nonetheless, it is important to consider the implications of using these types of dialogues when trying to address more female gender-sensitive issues in communities.

The perspectives of the migrants were captured in the action plans, especially because the LG officials and the JAC chairmen ensured that the agenda of these vulnerable groups (developed during the single stakeholder dialogues) were reflected in the plans. This highlights the difference between participation and representation. It suggests that there may be more efficient ways of carrying out such interactive processes to ensure that participation of vulnerable groups in meetings is not merely “window dressing”. This study makes a case for the use of ‘boundary spanners’—these are ‘elite’ individuals who can be a voice for the minorities and who could have the opportunity to influence decision making. For instance, the young women can set the priorities in single stakeholder dialogues and choose influential members of the community (such as teachers, social welfare workers, traditional leaders) who are sympathetic to their cause to represent them in dialogues within such patriarchal contexts. A review on community participation by Kenny et al. [36] noted that to move from ‘symbolic’ participation to shared decision making and co-production of knowledge requires early discussions with the communities regarding co-ownership of the process and the commitment needed. The authors noted that the use of key community leaders and community ‘champions’ was helpful in addressing the challenges of inclusion and representation [36].

Though a democratic process (voting) of decision making was followed and community members outnumbered the other stakeholders, negotiations which occurred during dialogues before voting were directed by knowledge, hierarchy, social status and cultural norms and these proved to be stronger influences. However, Goold et al. [37] found in their CBPR study that using a supermajority of minority and underserved community members (15 in number) compared to four representatives of research institutions helped to reduce power imbalance. Though this was a different context (North America), the intervention was targeted at marginalized ethnic groups. This finding suggests that the disparity in numbers between the groups may have to be much greater in favour of the less powerful party to produce an effect. However, Elberse et al. [23] also had a majority of care users (29) compared to 13 experts in their study and concluded that it led to more strategic defensive behaviour in the powerful.

In our study, though we adapted the framework by Elberse et al. [23] to suit our context, exclusion resulting from issues such as intra-community dynamics and local traditional structures could not be addressed readily. Additionally, intra-community dynamics whereby some people had access to further deliberations with the prime traditional rulers may have led to intended and /or unintended exclusion of the perceptions of some community members, especially the vulnerable groups. It may have helped that the topic was a non-controversial one and had the buy-in of the traditional and other structures, thereby still enabling positive outcomes. However, the strong traditional influence seen in this study provides some insight into the limitation of using CPBR within the context for topics that would challenge cultural norms. It also highlights that for different contexts and topics, researchers must be aware of limitations of who can participate and contribute, and have their ideas accepted. Goold et al. [37] described that each of the tribes in their CBPR had their own sovereignty, noting the need for a more intense approach towards respectful engagement to ensure better participation and representation ultimately leading to better outcomes.

### Key lessons learned

How do we then set up a dialogue process to allow better inclusion? What can be added to the framework by Elberse et al. to reduce exclusion further, especially within socio-cultural contexts similar to ours? Elberse et al. did not have single stakeholders dialogues but they recommended that the less vulnerable groups should come to the dialogues with a clear agenda. The single stakeholder dialogues enabled us to ensure that the priorities of the different groups were captured before the multi-stakeholder dialogues. Another important lesson learned is that if evidence is used in the dialogues, even when exclusion of certain individuals occurs, the outcomes could still favour them. It seems that representation of the vulnerable groups during the multi-stakeholder dialogues is also key in this regard. Special attention needs to be paid to intercommunity dynamics to enable different groups to formulate their views better; prepare for participation; and identify ‘boundary spanners’. It would be useful to note the representation of vulnerable groups that are readily accepted in the communities (e.g. migrants, young women) and representation of those not readily accepted (e.g. teenage mothers, people living with HIV/AIDS). To identify proper representation for these groups within communities, an initial step would be to use an external independent researcher to collect evidence, combined with single stakeholder dialogues of vulnerable groups to identify the norms, values and relevant issues. In essence, a successful process of change using the CBPR within the context would include: (1) a situational analysis as a starting point for dialogues; (2) Single stakeholder dialogues that would also enable identification of proper representation for vulnerable groups in the multi-stakeholder dialogues; and (3) Discussions on inclusion with key stakeholders to identify opportunities (“wiggle room”) in view of strong / rigid local authorities.

It is important with representation that the citizens (vulnerable groups) are also there—to ensure that it works. The presence of the vulnerable groups in the dialogues would also act as a check on their representatives. Eversole [38] suggests that it is not possible to fully represent people who are not participating directly. Nevertheless, she noted the value of using ‘translation agents’—individuals who were comfortable in interactions with both the powerful and the powerless and therefore were able to facilitate effectively engagements between the two groups [38]. In our study, some of the LG officials and community members could be described as such ‘translation agents’ or ‘boundary spanners’.

Some authors argue that not all authoritative influence is negative or motivated by selfish reasons—sometimes power is exerted for positive purposes and can propagate better performance in the health sector. Erasmus and Gilson [39] noted that judgements of the effects of the exercises of power should be rooted in the context in which power is exerted. In our context, LG officials and the JAC chairmen leveraged the power of the traditional rulers to achieve the goals of the dialogues. This finding is supported by the review by Kenny et al. [36] who described the benefit of engaging key community leaders especially in planning and implementation of issues relating to governance.

The framework by Elberse et al. [23] provided a systematic and structured way to develop useful inclusion approaches that enabled the channelling of stakeholder influences to produce positive outcomes and this represents an opportunity. Since the pre-dialogue workshop contributed to intergroup collaborations, future interactions may benefit from involving stronger political and traditional rulers in pre-dialogue workshops. This would increase knowledge and skills in the participatory approach and more understanding of the inclusive aim of the dialogues. This could ultimately lead to intentional empowerment of young women and other vulnerable groups by these strong traditional/political stakeholders. Additional strategies to empower young women such as economic support, literacy, etc. could also reinforce this.

In our CBPR, the researchers used evidence (situational analysis) to identify vulnerable groups and relevant people in the household and community decision-making networks. While we found this very useful, we are also aware that using empirical evidence to structure participation has its limitations. We may have missed some more implicit, informal structures of power within the communities. These are important points for reflection for us as researchers. We do not know if the choices made by the community leaders and local government based on our checklist led to nominations that resulted in biases. For instance, a TBA was chosen in Ilara but not in Ipara and this could have shaped the community action plans differently.

We recommend further research to understand how exclusion of less privileged community members can be avoided during multi-stakeholder dialogues in the presence of power asymmetries between stakeholder groups. More in-depth study of intra-community power dynamics especially within the single stakeholder dialogues may give more insight into exclusion. We also suggest more research to display the role of CBPR in different contexts and topics including sensitive issues.

## Conclusion

CBPR can enable local governments to develop effective partnerships with health workers and communities to achieve health related goals even in the presence of power asymmetries between stakeholder groups. Inclusion strategies in dialogues can improve participation and enable shared decision making, however exclusion of vulnerable groups may still occur. Intra-community dynamics and socio-cultural contexts can drive exclusion and less privileged community members require proper representation to enable their issues to be captured effectively. However even when boundary spanners are used to represent vulnerable groups, those community members still need to participate to ensure better inclusion.

## Supporting information

S1 File Summary results—Single group dialogues. (DOCX)

S2 File Topic guides. (DOCX)
