# Supplementary material for: Inclusion strategies in multi-stakeholder dialogues: The case of a community-based participatory research on immunization in Nigeria
Source: PLoS One. 2022 Mar 22;17(3):e0264304. doi: 10.1371/journal.pone.0264304 (PMC8939808; doi:10.1371/journal.pone.0264304)
Supplement: S1 File — (DOCX) [file pone.0264304.s001.docx]

# Additional File 1 - Summary Results – Single group dialogues

## Set up of the single dialogues

The dialogues took place first within the three stakeholder groups – Selected community members, each in Ipara and Ilara wards, health workers in their respective wards and Remo-North local government (LG) officials. The dialogues were facilitated by the research team. The community dialogues captured perspectives of the community women and men in single gender dialogue settings (in each ward). Then five women and five men were nominated from each group to represent the rest in the community dialogues (in each ward). The action plans developed and the issues raised during the single gender dialogues were tabled for discussions in the community dialogues. A common community action plan was developed for each ward.

In total, during the single group dialogues, the stakeholders developed five action plans: combined action plans of community men and women for each ward, plans by health workers in both wards and a plan by Remo LGA officials. All the dialogues were recorded and the processes observed and captured using observation checklists. Facilitators and observers had been trained earlier in the use of the checklists. Table 1 shows inclusion strategies used in the single group dialogues and what happened during the dialogues.

*Table 1: Inclusion strategies and the single group dialogues*

| **Inclusion Strategies by Category** | **Summary results** |
| --- | --- |
| **Circumstances**  Neutral venue accessible to all parties, away from the influence of traditional leaders and elders, where community members would feel free to express their opinions (i.e. a town hall, sitting arrangements, no function tags).  Clear explanations and instructions were given to participants on what to expect and the aims of the CBPR, as well as the need for everyone to listen and feel listened to | The use of a neutral venue did not make any difference to the dialogues of health workers and the LG officials. These two groups already worked together - they were already involved in the implementation of the Reach Every Ward (REW) strategy in both wards and in the local government area – so they were comfortable in their respective groups. Both groups quickly identified the contextual and programmatic issues and developed their action plans.  Overall, the use of a neutral venue did not prevent the feelings of exclusion among some community members especially for some of the young women who were still reticent. Even in the single female groups, the older women were more vocal than the young women especially in Ilara though the young women contributed to discussions. |
|  | |
| **Behaviour**  The dialogues aimed to provide equal opportunities to speak. Facilitators were instructed to stimulate input of community members, especially women.  A pre-dialogue workshop was held with the three groups to discuss the inclusive aim of the dialogues and action process and the need for community ownership in order to develop and implement sustainable local solutions; the power asymmetry between the three groups and the need to ensure that community members had equal opportunities to speak and felt listened to*.*  To address socio-cultural norms and gender relations which result in the women not speaking, single stakeholder dialogues with young and older women were held separately to capture their views before the dialogues held together with community men to develop the community action plans. | The health workers in Ilara described the participation of all the frontline health workers in the dialogues, issues were raised and discussed together in order to reach a conclusion. Similarly, their Ipara counterparts detailed their discussions on immunization issues and challenges, assessing their capacity and brainstorming to find solutions. The LG officials also described a similar process in their single group dialogues and noted that having the opportunity to brainstorm in this way was an ‘enjoyable’ exercise for them because of their knowledge of immunization issues. A moderator was chosen by each group of health workers or LG officials and decisions made according to the general consensus. An additional element described by an LG official was that the LG officials met after the joint group dialogues to discuss what had been said by the three groups in Ilara and Ipara and to evaluate and encourage themselves.  Several things had been put in place to ensure that the views of community women were sufficiently captured. During the single group dialogues, the women in each ward had their own dialogues and developed their own action plans. Next they had to choose representatives to team up with the men to develop the community action plan. They generally chose a mix of older and young women and aimed at ensuring that the more vocal women in the group represented them – for instance, women that had leadership roles in the community and were used to such interactions with men; an example is a Traditional Birth Attendant (TBA) in Ilara who also happened to be a woman leader. In addition, some young women were chosen since immunization was considered more relevant to this group. It was obvious during the first round of dialogues that they had to overcome the social norms of keeping quiet before their elders and the men, though the importance of everyone having a voice and how that would impact the success and acceptability of their plans and implementation of actions decided had been emphasized in the pre-dialogue workshops.  Older women also considered that some of the young women did not have enough knowledge about immunization and that the dialogues were an opportunity for them to learn: “*I can vividly see that (the diversity) is working really well because there are kids (young people) in our midst who did not have an idea of what was going on but we have come together and they have been enlightened as regards the development relating to immunization in the community. That is what I have observed.” - Older woman (TBA) – Ilara*  For the Ilara community dialogues, the community participants described a process of discussing and listening to each other. They emphasized that no one was looked down upon regardless of their status and age. In Ipara, the community participants expressed that the focus of their single group dialogue was to discuss issues related to immunization and find out the perception of the community on those issues. *“The dialogue is smooth because when we say up it is always up and if we mean down it is always down. We agree with each other’s opinions and we don’t look at how small a person may be and disobey him or her. We will crosscheck anything that has been said. There is nothing like we shouting or fighting. You can’t even hear any talk unless a contribution is being made or we are laughing, we are one.” – Older woman - PAR participant, Ilara*  Additionally, in the community group dialogues, some participants, usually 2-3 who were more educated or influential or more used to being part of committees like the Ward Development Committee (WDC) were more active in the discussions than others. Yet, the more passive ones still contributed to the discussions especially when asked directly. Contributions to discussions were partly determined by people’s experience and knowledge - the Igede representatives for instance, could contribute more relating to their group; the chairman in Ipara for instance could contribute a lot on the work done by the WDC, what was working or not; some other members could talk more about issues relating to the status of community members and their attitudes towards immunization.  There were some arguments during the prioritization of issues to be included in the common action plan (derived from the action plans) of the community men and women. The women single group dialogues in Ilara and Ipara had nominated women whom they considered could talk more boldly to be part of the dialogues with the men – so they were able to negotiate. |
|  | |
| **Verbal**  Dialogues involving the community members were all held in the local Yoruba language.    Pre-dialogue presentations emphasised the importance of the community members’ perspectives to all three groups of stakeholders. Research assistants were instructed to guard the input of the community members and ensure that they were captured accurately in the community and joint action plans. | Analysis of the observational check lists used in the single group dialogues confirmed the relative ease of discussions among the homogenous groups. Overall, health workers and LG participants within the single group dialogues were more comfortable with each other as already noted.  The use of the Yoruba language made communication easy for the community members – this may have reduced feelings of exclusion but socio-cultural behavioural issues outweighed this element. Nevertheless, for all the single groups, there was no language barrier and all the participants reported that they had not perceived that any of their ideas were ridiculed. |
