# Supplementary material for: Inclusion strategies in multi-stakeholder dialogues: The case of a community-based participatory research on immunization in Nigeria
Source: PLoS One. 2022 Mar 22;17(3):e0264304. doi: 10.1371/journal.pone.0264304 (PMC8939808; doi:10.1371/journal.pone.0264304)
Supplement: S2 File — (DOCX) [file pone.0264304.s002.docx]

# Topic Guide - In-depth interviews with PAR Participants

## Introduction

The overall questions to be answered during these discussions relate to perceptions of the PAR participants in Remo North LGA; how they perceive the PAR process that they have been involved in, their views of the process of consensus building, shifts in group dynamics, issues related to trust and trust building, perceived changes that have taken place in the relationships and interactions between the three groups of stakeholders involved in the PAR.

Total participant time: [60-90 mins]

*This is a general guide for the interviews..*

*Before the interview begins, read out the informed consent form and ask for permission to continue. Document informed consent after explaining the research and ensuring participant comprehension. Obtain the signature or thumbprint of the participant after obtaining permission to continue.*

*If verbal consent only can be obtained, write the date on your version of the consent form to document the consent date. Write the name of the participant on your version of the consent form.*

**Introduction: [Time]**

Good Morning/Afternoon Sirs/Madams, I am [name], working for the Ogun state PHCDB and the Royal Tropical Institute in the Netherlands. We are doing a research on **the use of participatory evaluation and action research to increase immunization utilization in Ogun state.**

My name is ….

We are conducting this research because we like to understand better what the PAR participants’ experiences are with the PAR study, how the relationship was between the community members, health workers and local government officials and how things were done. We intend to use your views to understand if and how the communities developing a plan together with health providers and government will enable them to work better together and to improve the use of immunization for different groups in the community. Your views are important and we appreciate your participation.

The **objectives** of this interview are:

1. To find out about the dynamics of the three groups involved in the PAR
2. To find out the changes that have taken place in the behaviours of the community members, health workers and local government officials involved in the PAR as a result of the PAR and especially in the last year
3. To find out the changes that have taken place already in the communities as a result of the PAR and especially in the last year
4. To understand the reasons for any changes that have taken place

You are invited to participate because we think you know a lot about this, because you participated in this study.

It is important that you share your points of view freely. There are no right or wrong answers. We are interested in hearing different perspectives and opinions. We hope you’ll feel free to speak openly and honestly. Our discussion will last for about one and half hours

If you don’t understand a question, please let me know. You may refuse to answer any questions or withdraw from the study at any time.

The information that you give us is completely confidential. We will not associate your name with anything that you say.

If it is okay with you, I would like to record this discussion. This is to ensure that I don’t miss any of your comments. Only the research team will have access to the tapes and they will be destroyed as soon as we have completed our study. Do we have your permission to record?

Do you have any questions? [Respond to questions]

If you have any questions now or after our discussion has ended, you can always talk to a study team member like me or contact the supervisor whose information is on the consent form you completed.

Do we have your permission to proceed with the discussions? *(Ensure that consent is obtained before proceeding)*

Before we begin, let’s learn a bit more about each other by going around the room and introducing ourselves. I’ll start.

## In-depth interviews with community members participating in the PAR

**Introductory Questions**

1. (*Establish Rapport*) Please could you tell me something about the different groups in this community? (**Probe** for men/women groups; groups that work on health/immunization like the WDC/SMC; community leadership; vulnerable groups)

- Where are these groups?
- How are group decisions made?
- Who would you regard as influential in decision making in the groups? Why? (Probe for the different groups)

1. How did you get selected to participate in the dialogues?

- Also how did you get selected to represent the community group in the collective group with the health workers and the local government?

**Group Dynamics and Social Networks related to the PAR and health/immunization**

1. How do you work together with the health workers at the moment?
2. How would you describe the relationship between the communities, health workers and the local government in this new arrangement that was put in place for immunization delivery? (**Probe** for group dynamics, what structures are in place eg MOUs etc. How diverse are the participants in the groups in terms of SES, gender distribution, religious and cultural beliefs, etc;?)

- How has this worked between and within the groups?

1. Please could you describe what happens when you meet together with each other about immunization issues?

- Within the community dialogues
- Between the three groups?

1. What do you think about participation in the dialogues?

- How often do you place items on the agenda for discussion during the dialogues? ( **Probe** **for leadership and participation**)
  - How easy is it for you to bring up new ideas or issues on the agenda?
  - When you want to bring up a new point, what happens?

1. In the past year, how would you describe the willingness to speak and express opinions at group meetings - has it increased, remained the same, or decreased?

- For you?
- For the group members?

(**Probe** **for comfort level for expressing opinions: communication** within the community group and in the group with the community members, health workers and local government stakeholders)

1. What do you think about the time it takes to reach decisions in the groups? (**Probe for decision-making procedures** within the community group and in the collective group with the three stakeholder types).

- How true is it that everyone in the groups has a voice in the decisions?
- How committed do you feel to decisions that are made by the groups?
- How satisﬁed are you with the way the decision-making process is working?
- How much do you feel pressured to go along with decisions of the groups even though you might not agree? (**Probe for level of inﬂuence and power of self and others in the group**) Ask for **examples.**
- Do you feel your opinion is listened to and considered by other group members? How do you know that people are listening to you?
- Do certain individuals have more inﬂuence over the agenda at group meetings than others? (***In all cases, probe for within the community group and in the collective group***)
- Do certain individuals have more inﬂuence over the decision-making process than others? What do you think about this? Why do you think this is easier for those people?
- Are you bothered that certain individuals have more inﬂuence over the decision-making process than others?
- Would you like to have more input regarding the allocation of the groups’ resources?

1. How much do group members listen to each other’s points of view, even if they might disagree?
2. In working together to solve problems, how well has the groups been able to identify the important issues and generate several possible solutions? (**Probe** **for how well the groups recognize and address conﬂicts and problems.** Ask about both the community group and the collective group).

- How satisﬁed are you with the way the group deals with problems that come up? Please can you give an **example**?
- In your opinion, what (if any) have been the major points of conﬂict or disagreement within the group? How well do you feel that these conﬂicts were handled by the groups? – Does it always happen like that or was that an exception?

1. If you have agreed upon something in the group, do you believe that others will do it? (**Probe** **for perceived level of trust** within the community group and in the collective group) Ask for **examples.**

- Over the past year, has the amount of trust between group members increased, remained the same, or decreased?
- In the next year, how much trust do you expect to see between group members?

1. How much do you feel a part of the group (like you belong to the group)? (**Probe for sense of ownership/belonging to the group: cohesion**)

- How much do you have a sense of ownership over what the group does?
- Has this changed from the beginning? How? Why?
- Do you sometimes think of severing your afﬁliation with the group? Which (one) s? Why? Why not?

**Relevance of the Communities working together with the local government and health workers in immunization (Whether the PAR approach enabled the adaptation of immunization activities to meet the needs of the targeted communities, health workers and local governments)**

1. In your opinion, what activities do you consider participatory (i.e where everybody took part)?

- Who participated? And in what way?

1. How would you describe the results of this way of working together?

- **Probe** for usefulness in the communities expressing their needs
- In your opinion are the communities heard by health workers and local governments? How? Why so you think this?
- **Probe** for active participation of different groups in terms of gender, socio-economic class etc

1. What do you think about the willingness of the three groups to play their roles?

**Effectiveness - The extent to which the intended outcomes have been achieved, and were there any unintended outcomes?**

1. How would you describe the value of this way immunization issues are addressed by the communities, health workers and local government? (**Probe** for **perceived effectiveness of the partnership at immunization, partnership and personal levels**) **Probe** for perceptions of usefulness/importance of what the three groups are doing, ask for reasons; **Probe** for perceived value of the collaboration for the community as a whole and for the marginalized and poorest groups )
2. Do you think the decisions made during the dialogues have been implemented as planned? Why? Why not?
3. How much change has there been in how the group works together since you joined the project?

- In the past year, would you say that the group members’ capacity to work well together has: increased, remained the same, decreased?
- How do you think this collaboration can be improved?

1. During this past year, how would you describe changes in vaccination services? (**Probe** for **Changes in:** Distance to fixed immunization sites; Distance to outreach/mobile sites**;** Reliability of immunization supplies**;** Availability of health workers who vaccinate**;** Cost of immunization – direct, transportation, indirect)
2. Looking back on this year, how would you describe changes in the community due to vaccination? (**Probe** for **Changes in:** Immunization seeking behaviour for all groups in the community; access of immunization activities especially to the poorest and most marginalized groups. **Probe** for changes in awareness of REW activities by the communities; capacity building of the communities in the participatory approach)
3. How can these changes been explained - what do you think has made it possible for these changes to happen? Why? What else?
4. What do you think has made it difficult for changes to happen? Why? What else?

**Efficiency - The extent to which the Participatory, evaluation and action approach has been implemented as intended?**

23. How did the PAR work?

-         Were there changes in the groups participating in the JAPs and their implementation?

-         Were activities implemented as planned?

-         How would you describe the availability of all you needed during the planning and action phase of this project? (**Probe** for resources (financial, expertise, time) - available in time and sufficiently?)

**24.** How would you describe the use of the developed Joint Action Plans? (**Probe** for the way the JAPs were developed and their use in planning for the subsequent immunization activities)

**25.** In what ways did the willingness (or non-willingness) of the three groups facilitate or hinder efficient implementation? (**Probe** for if partial implementation or total implementation of the JAP that was not successful due to unexpected barriers).

**Opportunities for Sustainability – The extent to which what may have been achieved using the PAR can be sustained after the withdrawal of external support.**

***In this section ask specifically for the positive changes as a result of the participatory approach that have been described by the respondents in the previous sections***

1. In your opinion, what are the things which can make these achievements continue working even if there is no outside help? **(Probe** for examples of the use of local resources/ capacities and /or networks that are (or can be) effectively used to sustain the achievements of the response**. Probe** for the extent to which the community feels as if the immunization planning and work belong to them? **Ask for examples** of how the community has demonstrated ownership and capacity to self-support in the project)
2. In your opinion, how is this partnership working (or not)?

- In your opinion how long do you expect that the behavioural changes among the health workers, local government and communities (**ask for each separately**) will last? Why?
- (*If working*) How long do you expect this kind of partnership among the health workers, local government and communities (**ask for each separately**) to work? Why?
- What do you think of introducing this way of working in other areasof health and other sectors? Why? Why not?

**Closing:**

Let’s take a moment to review our work. [Summary of work] This concludes the focus group. Thank you for your time and participation.

*Please add some information on characteristics of the group:*

**Background Characteristics:**

Age

Sex

Occupation

Ward/Community

Any key role played within the groups in the PAR

## In- Depth interview with health workers participating in the PAR

**Introductory Questions**

1. (*Establish Rapport*) Please could you tell me something about immunization services provided in this community? (**Probe** for the stakeholders at all levels including community structures/ committees that are involved in immunization services in this community; Probe for perception of access to services and information for different groups including vulnerable groups; **Probe** for immunization activities by health services, LGA, NGO etc)
2. In your opinion has the immunization programme been responsive to the needs of the community? How? Why? (ask specifically for the PAR approach)
3. How did you get selected to participate in the dialogues?

- How did you get selected to represent the health workers’ group in the collective group with the communities and the local government?

**Group Dynamics and Social Network related to immunization**

1. How do you work together with the communities at the moment?
2. How would you describe the relationship between the communities, health workers and the local government in this new arrangement that was put in place for immunization delivery? (**Probe** for group dynamics, what structures are in place eg MOUs etc. How diverse are the participants in the groups in terms of SES, gender distribution, religious and cultural beliefs, etc;?)

- How has this worked between and within the groups?

1. Please could you describe what happens when you meet together with each other about immunization issues?

- Within the health workers’ dialogues
- Between the three groups?

1. What do you think about participation in the dialogues?

- How often do you place items on the agenda for discussion during the dialogues? ( **Probe** **for leadership and participation**)
  - How easy is it for you to bring up new ideas or issues on the agenda?
  - When you want to bring up a new point, what happens?

1. In the past year, how would you describe the willingness to speak and express opinions at group meetings - has it increased, remained the same, or decreased?

- For you?
- For the group members?

(**Probe** **for comfort level for expressing opinions: communication** within the health workers’ group and in the collective group with the community members, health workers and local government stakeholders)

1. What do you think about the time it takes to reach decisions in the groups? (**Probe for decision-making procedures** within the health workers’ group and in the collective group with the three stakeholder types).

- How true is it that everyone in the groups has a voice in the decisions?
- How committed do you feel to decisions that are made by the groups?
- How satisﬁed are you with the way the decision-making process is working?
- How much do you feel pressured to go along with decisions of the groups even though you might not agree? (**Probe for level of inﬂuence and power of self and others in the group**) Ask for **examples.**
- Do you feel your opinion is listened to and considered by other group members? How do you know that people are listening to you?
- Do certain individuals have more inﬂuence over the agenda at group meetings than others? (***In all cases, probe for within the health workers’ group and in the collective group***)
- Do certain individuals have more inﬂuence over the decision-making process than others? What do you think about this? Why do you think this is easier for those people?
- Are you bothered that certain individuals have more inﬂuence over the decision-making process than others?
- Would you like to have more input regarding the allocation of the groups’ resources?

1. How much do group members listen to each other’s points of view, even if they might disagree?
2. In working together to solve problems, how well has the groups been able to identify the important issues and generate several possible solutions? (**Probe** **for how well the groups recognize and address conﬂicts and problems.** *Ask about both the health workers’ group and the collective group*).

- How satisﬁed are you with the way the group deals with problems that come up? Please can you give an **example**?
- In your opinion, what (if any) have been the major points of conﬂict or disagreement within the group? How well do you feel that these conﬂicts were handled by the groups? – Does it always happen like that or was it an exception?

1. If you have agreed upon something in the group, do you believe that others will do it? (**Probe** **for perceived level of trust** within the health workers’ group and in the collective group) Ask for **examples.**

- Over the past year, has the amount of trust between group members increased, remained the same, or decreased?
- In the next year, how much trust do you expect to see between group members?

1. How much do you feel a part of the group (like you belong to the groups)? (**Probe for sense of ownership/belonging to the group: cohesion**)

- How much do you have a sense of ownership over what the group does?
- Has this changed from the beginning? How? Why?
- Do you sometimes think of severing your afﬁliation with the groups? Which (one)s Why? Why not?

**Relevance of the Communities working together with the local government and health workers in immunization (Whether the PAR approach enabled the adaptation of immunization activities to meet the needs of the targeted communities, health workers and local governments)**

1. In your opinion, what activities do you consider participatory (i.e where everybody took part)?

- Who participated? And in what way?

1. How would you describe the results of this way of working together?

- **Probe** for usefulness in the communities expressing their needs
- In your opinion are the communities heard by health workers and local governments? How? Why so you think this?
- **Probe** for active participation of different groups in terms of gender, socio-economic class etc

1. What do you think about the willingness of the three groups to play their roles?

**Efficiency - The extent to which the Participatory, evaluation and action approach has been implemented as intended?**

1. How did the PAR work?

- Were there changes in the groups participating in the JAPs and their implementation?
- Were activities implemented as planned?
- How would you describe the availability of all you needed during the planning and action phase of this project? (**Probe** for resources (financial, expertise, time) - available in time and sufficiently?)

1. How would you describe the use of the developed Joint Action Plans? (**Probe** for the way the JAPs were developed and their use in planning for the subsequent immunization activities)
2. In what ways did the willingness (or non-willingness) of the three groups facilitate or hinder efficient implementation? (**Probe** for if partial implementation or total implementation of the JAP that was not successful due to unexpected barriers).

**Effectiveness - The extent to which the intended outcomes have been achieved, and were there any unintended outcomes?**

1. How would you describe the value of this way immunization issues are addressed by the communities, health workers and local government? (**Probe** for **perceived effectiveness of the partnership at immunization, partnership and personal levels**) **Probe** for perceptions of usefulness/importance of what the three groups are doing, ask for reasons; **Probe** for perceived value of the collaboration for the community as a whole and for the marginalized and poorest groups )
2. How much change has there been in how the group works together since you joined the project?

- In the past year, group members’ capacity to work well together has: increased, remained the same, decreased?
- How do you think this way of working together can be improved?

1. Looking back on this year, how would you describe changes in the health workers’ behaviour due to the participatory approach of working? (**Probe** for changes in skills and responsiveness of the health workers; REW strategy modifications due to the JAPs; capacity in participatory approach; ) How? Please give some **examples**
2. During this past year, how would you describe changes in vaccination services? (**Probe** for Changes in: Distance to fixed immunization sites; Distance to outreach/mobile sites; Reliability of immunization supplies; Availability of health workers who vaccinate; Cost of immunization – direct, transportation, indirect)
3. Looking back on this year, how would you describe changes in the community due to vaccination? (**Probe** for Changes in: Immunization seeking behaviour for all groups in the community; access of immunization activities especially to the poorest and most marginalized groups. **Probe** for changes in awareness of REW activities by the communities; capacity building of the communities in the participatory approach)
4. How can these changes been explained - what do you think has made it possible for these changes to happen? Why? What else?
5. What do you think has made it difficult for changes to happen? Why? What else?
6. In your opinion, have there been any unintended outcomes? If yes, please could you elaborate?

**Opportunities for Sustainability – The extent to which what may have been achieved using the PAR can be sustained after the withdrawal of external support.**

***In this section ask specifically for the positive changes that have been described by the respondent in the previous sections***

1. In your opinion, what are the things which can make these achievements continue working especially if there is no outside help? **(Probe** for examples of the use of local resources/ capacities and /or networks that are (or can be) effectively used to sustain the achievements of the response**)**
2. How would you describe the extent to which everybody involved has taken ownership of this project? **(Probe** for the PHCDB, MOH, LG, ward authorities, communities, community leaders and health workers – **ask for examples** of how each group has demonstrated ownership and capacity to self-support in the project)
3. In your opinion, how is this partnership working (or not)?

- In your opinion how long do you expect that the behavioural changes among the health workers, local government and communities (**ask for each separately**) will last? Why?
- (*If working*) How long do you expect this kind of partnership among the health workers, local government and communities (**ask for each separately**) to work? Why?
- What do you think of introducing this way of working in other areas of health or other sectors? Why? Why not?

**Closing:**

This concludes the interview. Thank you for your time and participation.

**BACKGROUND VARIABLES**

(To be filled in by the interviewer)

Male / Female

Age

Profession/Position

No of years of experience overall (and in this facility)

No of years of experience working in immunization

Any key role in the PAR process

## In-depth interview with local government officials participating in the PAR

**Introductory Questions**

1. (*Establish rapport*) How would you describe use of immunization services by the communities in this local government area?

- Are there differences for different groups? For the poor?

1. How do you ensure that everyone in the community is reached by the immunization interventions and has equal access to services and information, including the most vulnerable groups? (**Ask** for their opinion of who the vulnerable groups are; **Probe** for any special strategies for vulnerable groups. **Ask for examples)**
2. How did you get selected to participate in the dialogues?

- How did you get selected to represent the local government officials’ group in the collective group with the communities and the local government?

**Group Dynamics and Social Network related to immunization**

1. How do you work together with the communities at the moment?
2. How would you describe the relationship between the communities, health workers and the local government in this new arrangement that was put in place for immunization delivery? (**Probe** for group dynamics, what structures are in place eg MOUs etc. How diverse are the participants in the groups in terms of SES, gender distribution, religious and cultural beliefs, etc;?)

- How has this worked between and within the groups?

1. Please could you describe what happens when you meet together with each other about immunization issues?

- Within the local government officials’ dialogues
- Between the three groups?

1. What do you think about participation in the dialogues?

- How often do you place items on the agenda for discussion during the dialogues? ( **Probe** **for leadership and participation**)
  - How easy is it for you to bring up new ideas or issues on the agenda?
  - When you want to bring up a new point, what happens?

1. In the past year, how would you describe the willingness to speak and express opinions at group meetings - has it increased, remained the same, or decreased?

- For you?
- For the group members?

(**Probe** **for comfort level for expressing opinions: communication** within the local government group and in the collective group with the community members, health workers and local government stakeholders)

1. What do you think about the time it takes to reach decisions in the groups? (**Probe for decision-making procedures** within the local government group and in the collective group with the three stakeholder types).

- How true is it that everyone in the groups has a voice in the decisions?
- How committed do you feel to decisions that are made by the groups?
- How satisﬁed are you with the way the decision-making process is working?
- How much do you feel pressured to go along with decisions of the groups even though you might not agree? (**Probe for level of inﬂuence and power of self and others in the group**) Ask for **examples.**
- Do you feel your opinion is listened to and considered by other group members? How do you know that people are listening to you?
- Do certain individuals have more inﬂuence over the agenda at group meetings than others? (***In all cases, probe for within the local government group and in the collective group***)
- Do certain individuals have more inﬂuence over the decision-making process than others? What do you think about this? Why do you think this is easier for those people?
- Are you bothered that certain individuals have more inﬂuence over the decision-making process than others?
- Would you like to have more input regarding the allocation of the groups’ resources?

1. How much do group members listen to each other’s points of view, even if they might disagree?
2. In working together to solve problems, how well has the groups been able to identify the important issues and generate several possible solutions? (**Probe** **for how well the groups recognize and address conﬂicts and problems).** *Ask about both the local government group and the collective group*).

- How satisﬁed are you with the way the group (s) deals with problems that come up? Please can you give an **example(s)**?
- In your opinion, what (if any) have been the major points of conﬂict or disagreement within the group? How well do you feel that these conﬂicts were handled by the groups? – Does it always happen like that or was it an exception?

1. If you have agreed upon something in the group, do you believe that others will do it? (**Probe** **for perceived level of trust** within the local government group and in the collective group) Ask for **examples.**

- Over the past year, has the amount of trust between group members increased, remained the same, or decreased?
- In the next year, how much trust do you expect to see between group members?

1. How much do you feel a part of the group (like you belong to the groups)? (**Probe for sense of ownership/belonging to the group: cohesion**)

- How much do you have a sense of ownership over what the group does?
- Has this changed from the beginning? How? Why?
- Do you sometimes think of severing your afﬁliation with the groups? Which (one)s Why? Why not?

**Relevance of the Communities working together with the local government and health workers in immunization (Whether the PAR approach enabled the adaptation of immunization activities to meet the needs of the targeted communities, health workers and local governments)**

1. In your opinion, what activities do you consider participatory (i.e where everybody took part)?

- Who participated? And in what way?

1. How would you describe the results of this way of working together?

- **Probe** for usefulness in the communities expressing their needs
- In your opinion are the communities heard by health workers and local governments? How? Why so you think this?
- **Probe** for active participation of different groups in terms of gender, socio-economic class etc

1. What do you think about the willingness of the three groups to play their roles?

**Efficiency - The extent to which the Participatory, evaluation and action approach has been implemented as intended?**

1. How do you think the PAR worked?

- Were there changes in the groups participating in the JAPs and their implementation?
- Were activities implemented as planned?
- How would you describe the availability of all that was needed during the planning and action phase of this project? (**Probe** for resources (financial, expertise, time) - available in time and sufficiently?)

1. How would you describe the use of the developed Joint Action Plans? (**Probe** for the way the JAPs were developed and their use in planning for the subsequent immunization activities)
2. In what ways did the willingness (or non-willingness) of the three groups facilitate or hinder efficient implementation? (**Probe** for if partial implementation or total implementation that was not successful due to unexpected barriers).

**Effectiveness - The extent to which the intended outcomes have been achieved, and were there any unintended outcomes?**

1. How would you describe the value of this way immunization issues are addressed by the communities, health workers and local government? (**Probe** for **perceived effectiveness of the partnership at immunization, partnership and personal levels**) **Probe** for perceptions of usefulness/importance of what the three groups are doing, ask for reasons; **Probe** for perceived value of the collaboration for the community as a whole and for the marginalized and poorest groups )
2. Do you think the decisions made during the dialogues have been implemented as planned? Why? Why not?
3. How much change has there been in how the group works together since you joined the project?

- In the past year, would you say that the group members’ capacity to work well together has: increased, remained the same, decreased?
- How do you think this collaboration can be improved?

1. Looking back on this year, how would you describe changes in the local government’s behaviour due to the participatory approach of working? (**Probe** for changes in skills and responsiveness of the health workers; REW strategy modifications due to the JAPs; capacity in participatory approach; ) How? Please give some **examples**
2. During this past year, how would you describe changes in vaccination services? (Probe for Changes in: Distance to fixed immunization sites; Distance to outreach/mobile sites; Reliability of immunization supplies; Availability of health workers who vaccinate; Cost of immunization – direct, transportation, indirect)
3. Looking back on this year, how would you describe changes in the community due to vaccination? (Probe for Changes in: Immunization seeking behaviour for all groups in the community; access of immunization activities especially to the poorest and most marginalized groups. Probe for changes in awareness of REW activities by the communities; capacity building of the communities in the participatory approach)
4. How can these changes been explained - what do you think has made it possible for these changes to happen? Why? What else?

- What do you think has made it difficult for changes to happen? Why? What else?
- In your opinion, have there been any unintended outcomes? If yes, please could you elaborate?

**Opportunities for Sustainability – The extent to which what may have been achieved using the PAR can be sustained after the withdrawal of external support.**

***In this section ask specifically for the positive changes that have been described by the respondent in the previous sections***

1. In your opinion, what are the things which can make these achievements continue working especially if there is no outside help? **(Probe** for examples of the use of local resources/ capacities and /or networks that are (or can be) effectively used to sustain the achievements of the response**)**
2. How would you describe the extent to which everybody involved has taken ownership of this project? **(Probe** for the PHCDB, MOH, LG, ward authorities, communities, community leaders and health workers – **ask for examples** of how each group has demonstrated ownership and capacity to self-support in the project)
3. In your opinion, how is this partnership working (or not)?

- In your opinion how long do you expect that the behavioural changes among the health workers, local government and communities (**ask for each separately**) will last? Why?
- (*If working*) How long do you expect this kind of partnership among the health workers, local government and communities (**ask for each separately**) to work? Why?
- What do you think of introducing this way of working in other areas of health or other sectors? Why? Why not?

1. In your opinion, what is the receptivity of the PAR process in immunization service delivery at all three levels of government - federal, state and local levels?

- How can receptivity be ensured and sustained at all three levels?
- Who are the relevant stakeholders to engage?

**Closing:**

This concludes the interview. Thank you for your time and participation.

**BACKGROUND VARIABLES**

(To be filled in by the interviewer)

Male / Female

Age

Profession/Position

Any key role played in the PAR

No of years of experience in the health/immunization sector.
